# Role of *VHL*, *HIF1A* and SDH on the expression of miR-210: Implications for tumoral pseudo-hypoxic fate

**DOI:** 10.18632/oncotarget.14265

**Published:** 2016-12-27

**Authors:** Anna Merlo, Cristóbal Bernardo-Castiñeira, Inés Sáenz-de-Santa-María, Ana S Pitiot, Milagros Balbín, Aurora Astudillo, Nuria Valdés, Bartolomé Scola, Raquel Del Toro, Simón Méndez-Ferrer, José I Piruat, Carlos Suarez, María-Dolores Chiara

**Affiliations:** ^1^ Otorhinolaryngology Service, Hospital Universitario Central de Asturias, Instituto Universitario de Oncología del Principado de Asturias, Universidad de Oviedo, CIBERONC, Oviedo, Spain; ^2^ Service of Molecular Oncology, Hospital Universitario Central de Asturias, Instituto Universitario de Oncología del Principado de Asturias, Universidad de Oviedo, Oviedo, Spain; ^3^ Service of Pathology, Hospital Universitario Central de Asturias, Instituto Universitario de Oncología del Principado de Asturias, Universidad de Oviedo, Oviedo, Spain; ^4^ Service of Endocrinology and Nutrition, Hospital Universitario Central de Asturias, Instituto Universitario de Oncología del Principado de Asturias, Universidad de Oviedo, Oviedo, Spain; ^5^ Otorhinolaryngology Service, Hospital Gregorio Marañón, Madrid, Spain; ^6^ Stem Cell Niche Pathophysiology Group, Centro Nacional de Investigaciones Cardiovasculares, Madrid, Spain; ^7^ Department of Cardiovascular Physiopahology, Instituto de Biomedicina de Sevilla (IBiS), Hospital Universitario Virgen del Rocío, CSIC, Universidad de Sevilla, Sevilla, Spain; ^8^ Stem Cell Institute and Department of Haematology, University of Cambridge and National Health Service Blood and Transplant, Cambridge Biomedical Campus, UK

**Keywords:** succinate dehydrogenase, hypoxia inducible factor, von hippel lindau, paragangliomas, miR-210

## Abstract

The hypoxia-inducible factor 1α (HIF-1α) and its microRNA target, miR-210, are candidate tumor-drivers of metabolic reprogramming in cancer. Neuroendocrine neoplasms such as paragangliomas (PGLs) are particularly appealing for understanding the cancer metabolic adjustments because of their associations with deregulations of metabolic enzymes, such as succinate dehydrogenase (SDH), and the von Hippel Lindau (*VHL*) gene involved in HIF-1α stabilization. However, the role of miR-210 in the pathogenesis of *SDH*-related tumors remains an unmet challenge. Herein is described an *in vivo* genetic analysis of the role of *VHL*, *HIF1A* and *SDH* on miR-210 by using knockout murine models, siRNA gene silencing, and analyses of human tumors. HIF-1α knockout abolished hypoxia-induced miR-210 expression *in vivo* but did not alter its constitutive expression in paraganglia. Normoxic miR-210 levels substantially increased by complete, but not partial, *VHL* silencing in paraganglia of knockout *VHL*-mice and by over-expression of p76del-mutated pVHL. Similarly, *VHL*-mutated PGLs, not those with decreased *VHL*-gene/mRNA dosage, over-expressed miR-210 and accumulate HIF-1α in most tumor cells. Ablation of SDH activity in *SDHD*-null cell lines or reduction of the SDHD or SDHB protein levels elicited by siRNA-induced gene silencing did not induce miR-210 whereas the presence of *SDH* mutations in PGLs and tumor-derived cell lines was associated with mild increase of miR-210 and the presence of a heterogeneous, HIF-1α-positive and HIF-1α-negative, tumor cell population. Thus, activation of HIF-1α is likely an early event in VHL-defective PGLs directly linked to VHL mutations, but it is a late event favored but not directly triggered by SDHx mutations. This combined analysis provides insights into the mechanisms of HIF-1α/miR-210 regulation in normal and tumor tissues potentially useful for understanding the pathogenesis of cancer and other diseases sharing similar underpinnings.

## INTRODUCTION

A defining hallmark of cancer is metabolic reprograming. Most of cancer cells use glycolysis-based metabolism for proliferation and reprogramming of the tricarboxylic acid cycle and mitochondrial oxidative phosphorylation to adapt to challenging conditions [1]. Aside this general cancer feature, there is a wealth of evidences proving that altered metabolic enzymes and their metabolites are also oncogenic and not simply a product of tumor proliferation [2]. This is particularly relevant in pheochromocytomas and paragangliomas (PCC/PGLs) which frequently develop in patients with germline mutations of genes encoding the succinate dehydrogenase mitochondrial complex II (SDH). These associations provide a useful tumor model to unravel the molecular connections between mitochondrial activity and cancer. Importantly, besides PCC/PGLs, SDH inactivation is increasingly observed in other neuroendocrine malignancies as well as in gastrointestinal neoplasms and renal cell carcinomas [3], and, thus, it is expected that it will acquire a more general clinical relevance in cancer pathogenesis.

PGLs are rare neuroendocrine tumors of the autonomous nervous system. Although the majority of PGLs occur sporadically, about 30-45% of these tumors are hereditary due to germline mutations in the succinate dehydrogenase *SDHB, SDHC, SDHD, SDHA*, and *SDHAF2* genes (hereafter *SDHx* genes), among other predisposing genes [4]. Mutations in any of the *SDHx* genes have been associated to succinate accumulation. *In vitro* assays revealed that excess of intracellular succinate may lead to increased transcriptional activity of hypoxia-inducible factors (HIF) [5, 6] and up-regulation of pro-tumorigenic HIF target genes, such as carbonic anhydrase 9 (*CA9*) and hypoxia-inducible factor prolyl hydroxylase 3 (*EGLN3*), irrespective of oxygen levels (a phenomenon defined as ‘pseudohypoxia’). PGLs may also associate with germline mutations in the von Hippel-Lindau (*VHL*) tumor suppressor [7]. Inherited mutations in the *VHL* gene cause the VHL disease, an autosomal-dominant neoplastic disease that is associated with various tumour types, including clear cell renal cell carcinomas, haemangioblastomas, pancreatic neuroendocrine tumours and PCC/PGLs [8]. VHL type 1 disease is characterized by development of clear cell renal cell carcinomas and hemangioblastomas, but not PCCs, and is associated with gross deletions in *VHL*. Type 2 disease is predominantly associated with *VHL* missense mutations and the development of PCCs (type 2C) or PCCs and hemangioblastomas (type 2A) or PCCs, hemangioblastomas and RCCs (type 2B). Somatic *VHL* mutations (i.e. mutations in tumor but not germline DNA) have been also described in about 9% of PGLs [9–11]. Intriguingly, the two types of VHL somatic mutations (type 1 and type 2) have been identified in parasympathetic PGLs [11] and are thus thought to be involved in the development of these tumors via a molecular mechanism not completely understood. VHL functions as the substrate recognition component of an E3-ubiquitin ligase, which targets HIF for proteasomal degradation under normoxic conditions [12]. Thus, HIF represent the molecule where SDH and VHL dysfunctions converge in PGLs. Nonetheless, the role of HIF in SDH-related tumorigenesis remains controversial. Some reported transcription profiles have shown that *SDHx*-mutated PGLs (*SDHx*-PGLs) showed enrichment in genes associated with the HIF-pathway and accumulate HIF-1α [13, 14] whereas others did not find such an association thus arguing against the pseudohypoxia hypothesis in *SDHx*-PGLs [11, 15, 16]. In contrast to *SDHx*-PGLs, the canonical HIF signaling pathway has been more clearly detected in most tumors that carry *VHL* mutations [11].

Studies in *VHL*-related PGLs (*VHL*-PGLs) revealed that the HIF-pathway involves, among other cues, the HIF-1α-microRNA target, miR-210 [11, 16]. Importantly, *VHL*-PGLs, in addition to accumulate HIF-1α, express low levels of SDHB likely as a result of miR-210 up-regulation which also represses expression of other genes essential for mitochondrial metabolism [17]. This suggests that miR-210 is a potential oncomiR in *VHL*-deficient tumors which impact on mitochondria metabolism [16]. Thus, understanding of the *in vivo* mechanisms of miR-210 expression should increase our knowledge on cancer pathogenesis and allow the identification of cancer-specific vulnerabilities that could be exploited therapeutically. However, the role of miR-210 in the pathogenesis of *SDH*-related tumors remains an unmet challenge. These premises encouraged us to carry out an in-depth analysis of the role of HIF-1α, SDH, and VHL on miR-210 expression by using knockout murine models and analyses of human tumor material.

## RESULTS

### *In vivo* roles of VHL and HIF-1α on the expression of miR-210

**Figure 1:**
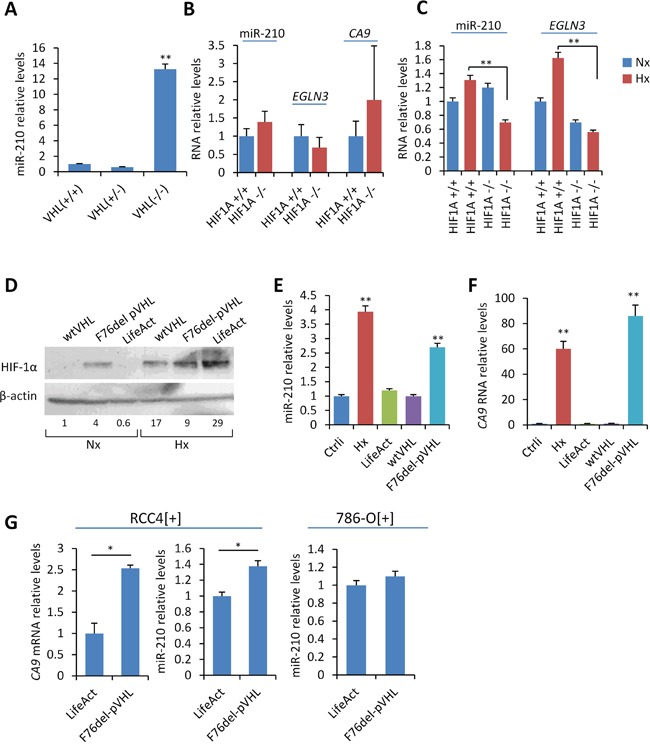
In vitro and in vivo analysis of the role of VHL and HIF-1α on miR-210 expression **A, B, C**. miR-210, *CA9* and *EGLN3* RNA levels were quantified by RT-qPCR in adrenal gland obtained from the indicated mice genotypes. For analysis of *HIF1A* knockouts, *HIF1A* +/+ and *HIF1A* −/− mice were exposed to hypoxia (10% O_2_) for 30 days prior to isolation of adrenal glands (panel C, hypoxia) or left under atmospheric O_2_ conditions (panel B, normoxia). **D**. Western blot showing HIF-1α protein levels upon ectopic expression of F76del VHL or wild type VHL in SCC40 cells incubated under normoxic (Nx: 21% O_2_) or hypoxic (Hx: 1% O_2_) conditions for 24 hours. Cells transiently transfected with LifeAct plasmid were used as controls (mock). β-actin, total protein-loading control. Fold change of HIF-1α protein levels upon normalization to total protein expression is shown below the Western blot image. **E, F, G**. miR-210 and *CA9* RNA relative levels in SCC40 (E, F) and RCC4[+] or 786-O[+] cells (G) treated as indicated in (D). *CA9* mRNA levels were undetectable in 786-O[+] cells even when incubated under hypoxic conditions. * P < 0.05 ** indicates P < 0.01.

We first addressed whether the expression of miR-210 in the paraganglionic system is regulated by VHL and/or HIF-1α activity *in vivo*. To this end, we analyzed miR-210 levels in tissues isolated from mice with either *VHL* or *HIF1A* conditionally deleted in the neural crest derived cells. We present here data obtained from adrenal medulla. Figure 1A shows a significant 13-fold increase of miR-210 levels in the adrenal gland of *VHL*-null mice in comparison with wild type mice. However, miR-210 levels did not change in heterozygous tissues obtained from *VHL* +/- thus indicating that a single functional copy of *VHL* produce enough functional VHL. In contrast to *VHL*, absence of HIF-1α did not alter the constitutive expression of miR-210. As shown in Figure 1B, miR-210 levels in the adrenal gland of normoxic knockout *HIF1A* −/− or *HIF1A* +/- mice were similar to those of wild type *HIF1A* +/+. Similarly, mRNA levels of other HIF-1α-targets, *EGLN3* and *CA9*, did not decrease in the absence or partial loss of HIF-1α. To further analyze the role of HIF-1α on miR-210 expression, wild type *HIF1A* +/+ and knockout *HIF1A* −/− mice were exposed to hypoxia (10% O_2_) for 30 days prior to organ isolation to allow for HIF-1α accumulation in tissues. Under these conditions, the functional inactivation of HIF-1α significantly reduced hypoxic expression of miR-210 as well as *EGLN3* in the adrenal medulla (Figure 1C).

Given that most tumors carrying *VHL* inactivating mutations are not necessarily accompanied by loss of heterozygosity, we also analyzed the impact of a *VHL* cancer-associated mutation, F76del VHL, on miR-210 expression by using SCC40 cells which, endogenously, express wild type VHL [18, 19]. F76del mutant was selected because represents the *VHL* gene alteration (c.227_229delTCT) associated with a wide spectrum of cancers including PGLs and no-neuroendocrine neoplasms related to VHL disease. We first used a squamous cell carcinoma cell line, SCC40, which has been well-characterized at the genetic and functional level with regard to HIF signaling [18, 19]. SCC40 cells were transfected with a *VHL*-expressing construct encoding for F76del-VHL. As controls, we used a *VHL*-expressing construct containing the wild type gene (wtVHL) or a LifeAct construct expressing an actin binding peptide (mock). Figure 1D shows that HIF-1α protein was not detected in mock-transfected cells incubated under normoxic conditions. By contrast, HIF-1α accumulated under normoxic conditions in cells transfected with F76del-VHL with further increase upon hypoxic treatment (1% O_2_, 24 h). As expected, normoxic accumulation of HIF-1α was not observed by ectopic expression of wild type-*VHL*. Consistent with our previously reported findings in PGL tissues carrying mutant F76del-pVHL [11], a ~ 3- and ~ 90-fold increase of miR-210 and *CA9* RNA levels, respectively, was detected upon ectopic expression of F76del-VHL under normoxic conditions as compared with wild type *VHL* or mock-transfected cells (Figure 1E and 1F). Because SCC40 cells endogenously express wild type *VHL*, these data show that a mutant non-functional F76del-VHL may abolish ubiquitination of HIF-1α by wild type VHL thus exerting a dominant negative effect that lead to the activation of the HIF-1/miR-210 pathway.

The above results were replicated in RCC4 renal carcinoma cells expressing wild type VHL (RCC4[+]). In the absence of a human cell model suitable for *in vitro* analysis of PGLs, cancer cells derived from clear cell renal cell carcinomas are valuable tools for studies of parasympathetic PGLs because of the genetic similarities between both types of tumors [11]. As shown in Figure 1G, a significant increase of miR-210 and *CA9* was detected upon expression of the F76del-VHL mutant in RCC4[+] cells. This result was not replicated in 786-O[+] cells that express wild type *VHL* and the HIF-2α rather than HIF-1α subunit thus suggesting that HIF-1α is the predominant HIF-α subunit involved in the pseudohypoxic up-regulation of miR-210.

### Roles of *SDHD* and *SDHB* on the expression of miR-210

**Figure 2:**
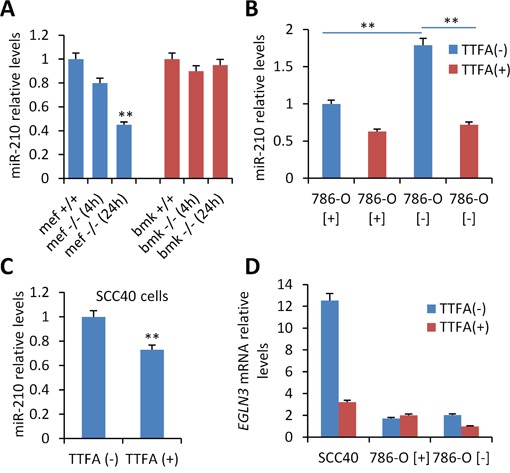
Analysis of the role of SDHD on miR-210 expression **A**. Mouse embryonic fibroblasts (MEFs) and baby mouse kidney (BMK) cells obtained from *SDHD*-ESR −/− mice and their homozygous *SDHD* flox/+ (+/+) and heterozygous *SDHD* flox/− (+/−) littermates were cultured in medium supplemented with 4-hydroxy-tamoxifen for 24 hours. **B, C, D**. *VHL*-proficient (786-O[+]) and *VHL*-deficient (786-O[-]) (B, D) or SCC40 cells (C, D) were incubated with 0.5 mM TTFA (TTFA(+)) or solvent (TTFA(-)) for 16 hours prior to miR-210 (B, C) or *EGLN3* (G) quantification by RT-qPCR. Values are expressed as mean ratios ± SD **, *P* <0.01, unpaired *t* test.

To determine whether *SDH* loss of function exert any effect on miR-210 expression, we used previously reported established cell lines (MEF and BMK) obtained from a tamoxifen-inducible *SdhD* knockout mutant mouse [20]. Homozygous *SdhD* −/−, but not heterozygous *SdhD* +/-, MEF and BMK cell lines are known to partially recapitulate the HIF pathway in comparison to wild type homozygous *SdhD* +/+ cell lines. By contrast, tissues (such as adrenal gland) derived from conditional homozygous *SdhD* −/− mice do not over-express HIF-1α nor its targets [20] and therefore they were not expected to over-express miR-210. Analysis of miR-210 in MEF and BMK cells revealed that ablation of the two (homozygous *SdhD* −/−) or one (heterozygous *SdhD* +/-) SdhD alleles failed to induce miR-210 over-expression in BMK-cells and even caused a reduction of miR-210 expression levels in MEF cells (Figure 2A). These data contrasted with the reported link between *SDHD* deficiency and HIF-1α accumulation what prompted us to use another cell-system to clarify the role of decreased mitochondrial complex II activity on miR-210 expression. We first used thenoyltrifluoroacetate (TTFA) which binds to the ubiquinone docking sites and abrogates the electron flow at the SDH complex. Figure 2B shows that inhibition of the succinate:ubiquinone oxidoreductase activity by 0.5 mM TTFA did not induce miR-210 over-expression in VHL-deficient (786-O[−]) or VHL-proficient (786-O[+]) cells. On the contrary, TTFA significantly reduced miR-210 levels in *VHL*-deficient cells. 786-O cells are known to mainly express HIF-2α subunit (although minimal levels of HIF-1α protein have been detected under hypoxic conditions [11]). Thus, we also tested the effect of TTFA on SCC40 cells which express HIF-1α subunit. As shown in Figure 2C, TTFA treatment did not induce miR-210 expression but slightly, although significantly, reduced it in SCC40 cells. Decreased mRNA levels were also detected for *EGLN3*, another HIF-target gene, in SCC40 cells treated with TTFA and in 786-O[-] cells (Figure 2D).

**Figure 3:**
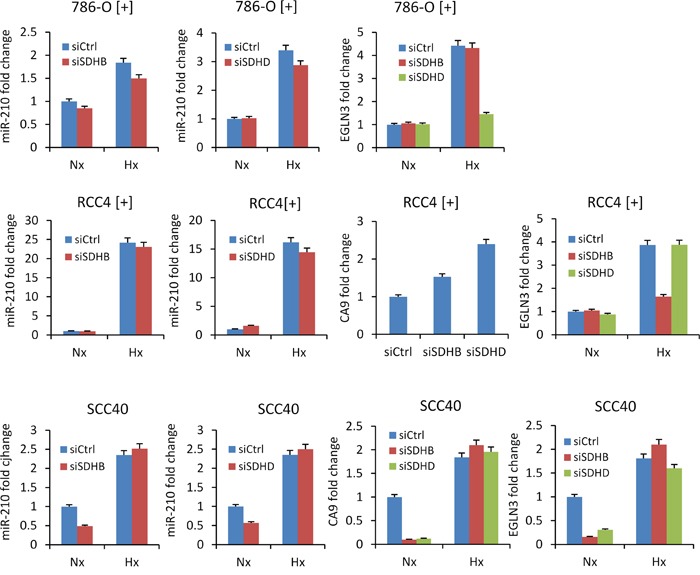
Absence of miR-210 up-regulation by siRNA-mediated silencing of SDHB or SDHD miR-210, *CA9* and *EGLN3* RNA levels were analyzed in the indicated cells following treatment with SDHD (siSDHD), SDHB (siSDHB) or control (siCtrl) siRNAs. 24 hours after transfection, cell were incubated under normoxic (Nx) or hypoxic (Hx, 1% O_2_ for 25 hours) conditions. Values are expressed as mean ratios ± SD.

**Figure 4:**
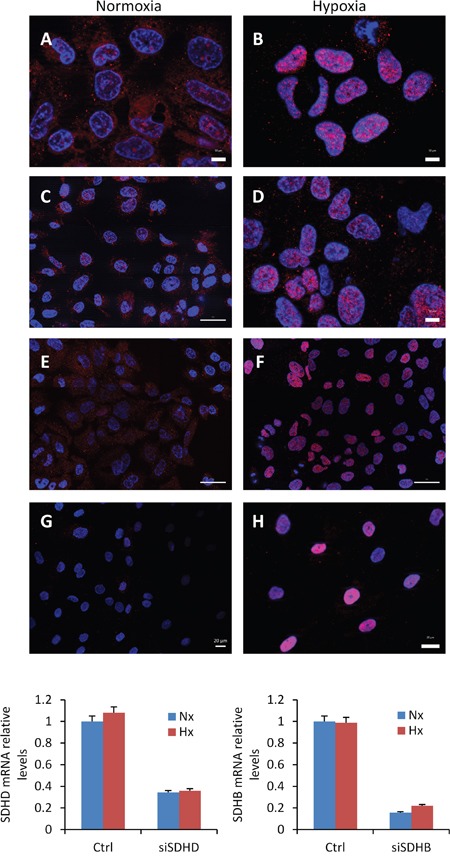
Absence of HIF-1α protein accumulation in nuclei of cells treated with SDHB or SDHD-siRNAs Immunocytochemical analysis of HIF-1α in SCC40 cells treated with Ctrl- **(A, B)**, SDHB- **(C, D)**, or SDHD- **(E, F)** -siRNAs and exposed to normoxia (A, C, E) or hypoxia (1% O_2_ for 24 h) (B, D, F). (G, H) Immunocytochemical analysis of HIF-1α in tumor associated fibroblasts derived from human PGL carrying *SDHD* mutation (*SDHD*-TAFs) and incubated under normoxic **G**. or hypoxic (1% O_2_ for 24 h) **H**. conditions; note the higher intensity of HIF-1α immunostaining (pink) in hypoxic *SDHD*-TAF in comparison with siSDHB- or siSDHD-treated SCC40 cells. Quantification of the SDHB or SDHD mRNA levels shows significant reduction of the mRNA levels after siRNA treatments.

The role of the SDH complex on miR-210 expression was further explored in cultured cell lines by using siRNAs targeting the *SDHD* or *SDHB* genes. For this analysis, we used cells expressing wild type VHL such as 786-O[+] cells, which mainly express HIF-2α subunit, and RCC4[+] and SCC40 cells, which mainly express HIF-1α subunit. Endogenous *SDHD* and *SDHB* mRNA levels were found to be 40% to 90% lower in cells transfected with either of the two siRNAs (siSDHB or siSDHD) as compared to control siRNA (siCtrl)-transfected cells (data not shown). However, increased miR-210 levels were not found in any of the transfected cells (Figure 3), thus suggesting that full SDH function is not required for HIF-α-independent miR-210 expression. The same experimental strategy was then applied but using cells that were incubated under hypoxic conditions (1% O_2_ for 48 h) after siRNA transfection to allow for HIF-α-dependent miR-210 over-expression (Figure 3). As expected, increased levels of miR-210 were detected in hypoxic versus normoxic conditions. However, siSDHD or siSDHB did not further increase the hypoxic effect on miR-210 levels. Analysis of *CA9* and *EGLN3* mRNA levels also revealed that other HIF-α-target genes were not induced by decreased SDHD or SDHB expression either in normoxia or in hypoxia. Only a subtle inductive effect was found for *CA9* in RCC4 [+] cells (*CA9* gene could not be analyzed in 786-O[+] cells because its mRNA was not detected neither in normoxia nor in hypoxia). In agreement with those data, immunocytochemical analysis of HIF-1α revealed nuclear accumulation of this protein in siSDHB- or siSDHD-transfected cells incubated under hypoxic conditions but not under normoxic conditions (Figure 4).

**Figure 5:**
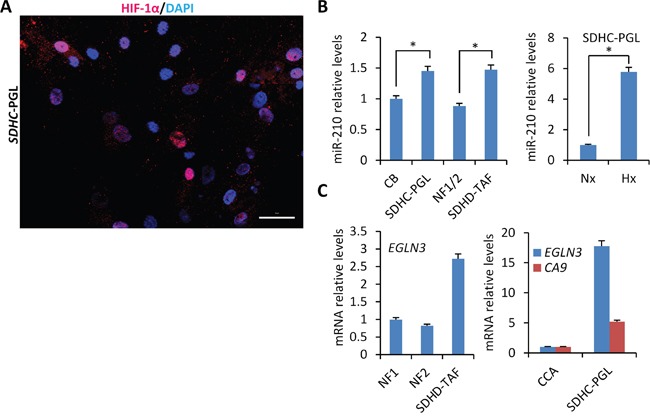
Mild heterogeneous activation of HIF-1α/miR-210 signaling axis in PGL-derived cells carrying SDHx mutations **A**. Immunocytochemical analysis of HIF-1α in cells derived from human PGL carrying SDHC exon 2 deletion (*SDHC*-PGL). Note the heterogeneity of the *SDHC*-PGL cell line which is composed of HIF-1α-positive and -negative cells. **B** and **C**. miR-210 (B), *EGLN3* and *CA9* **(C)** mRNA levels were analyzed by RT-qPCR in *SDHC*-PGL or *SDHD*-TAFs and compared with those of human normal carotid body (CB) or normal fibroblasts (NF), respectively. Two NF cell lines (NF1 and NF2) were used in this analysis. NF1/2 refers to the miR-210 data (mean ± SD value) obtained in NF1 and NF2 cells. *CA9* mRNA levels were undetectable in NF and TAF cells. Values are expressed as mean ratios ± SD.

The above data shows that HIF-1α accumulation is not triggered, at least in a significant and detectable level, by absence of SDHD activity or decreased SDHB or SDHD protein levels. We next tested whether HIF-1α accumulation could be associated with the presence of *SDHx* mutations instead of absence/decreased protein levels. All of *SDHx* genes are tumor suppressors, showing loss of heterozygosity, the loss of the normal allele in the tumors, in conjunction with the germline mutation. To analyze the impact of *SDHx* cancer-associated homozygous mutations on the expression of HIF-1α and miR-210, we used two PGL-derived cell lines, one composed of tumor cells which had been obtained from a tympanic PGL with homozygous *SDHC* exon 2 deletion (*SDHC*-PGL) [16] and other composed of tumor-associated fibroblasts which were derived from a *SDHD*-mutated (c.88_314+247del, p.His30Asn fs*11) jugular PGL (*SDHD*-TAFs). Thus, *SDHC*-PGL cells lack wild type SDHC protein whereas *SDHD*-TAFs express mutated and wild type *SDHD* gene (data not shown). Analysis of HIF-1α protein expression revealed that the *SDHC*-mutated cell line contains mixed populations of tumor cells, i.e. 72% were negative for HIF-1α protein expression but about 28% of cells accumulate nuclear HIF-1α even under normoxic conditions (Figure 5A). No HIF-1α accumulation was observed, however, in *SDHD*-TAFs in normoxia but they did accumulate higher HIF-1α levels, under hypoxic conditions, than the siSDHB or siSDHD-treated SCC40 cells thus suggesting that they are primed for a more intense HIF-1α-related cellular response (Figure 4). Figure 5B shows that both types of cells, *SDHC*-PGLs and *SDHD*-TAFs, have slightly but significant higher levels of miR-210 expression in normoxia than human paraganglia (normal carotid body) or non-tumoral fibroblasts, respectively. Analysis of *EGLN3* mRNA showed ~ 3-fold and ~ 18-fold higher levels in *SDHD*-TAF and *SDHC*-PGLs than in normal fibroblasts or carotid body, respectively (Figure 5C). Thus, in contrast to the data obtained in the *SDHD* knock-out mouse model or in the cell-based assays, cells carrying *SDHC* or *SDHD* mutations express higher levels of HIF-1α protein and its targets, miR-210, *CA9* and *EGLN3* than their normal counterparts. Nevertheless, the effect of mutations on HIF activation, if anything, seems to be rather subtle as compared with the stronger effect exerted by hypoxia (Figure 5B).

Collectively, the data indicates that the constitutive expression of miR-210 requires a functional wild type VHL protein but it is not influenced by partial *VHL* losses. HIF-1α, in turn, is involved in the hypoxic-induction of miR-210 but do not participate in its constitutive expression. Deleterious *VHL* gene mutations seems to have dominant negative effects on wild type VHL protein and as such activate the HIF-1α pathway. Finally, loss of *SDHD* or decreased *SDHB* or *SDHD* expression levels does not induce the HIF-1α/miR-210 pathway. Instead, mutations of *SDHx* genes may exert subtle inductive effects on the HIF-1α/miR-210 pathway.

### Pseudo-hypoxic fate of *VHL-* and *SDHx*-related PGLs

The above data cannot be directly extrapolated to the biology of tumor tissues inasmuch as they all represent cell-based models that do not necessarily recapitulate cell pathophysiology of PGLs. Thus, we next sought to determine whether the genetic-phenotypic correlations identified in the murine and cellular models were also observed in tumor tissues. To this end, we used a cohort of PGLs of parasympathetic origin with known *VHL* and *SDH* gene mutations. Germline mutations of *SDHD* or *SDHB*, and somatic heterozygous mutations of *VHL* were found in 11, 7, and 2 patients, respectively, while 25 patients did not carry *SDHx* and *VHL* somatic/germline mutations. *VHL* expression data was unknown for most of those tumors. Thus, we first performed a comprehensive analysis of the *VHL* gene by examining *VHL* DNA copy number and mRNA levels. Because, unexpectedly, we had previously identified somatic *VHL* gene mutations in parasympathetic PGLs [11], analysis of *VHL* gene dosage was also performed even though chromosomal losses on the 3p have not been previously described as frequent events in PCCs/PGLs [21].

**Figure 6:**
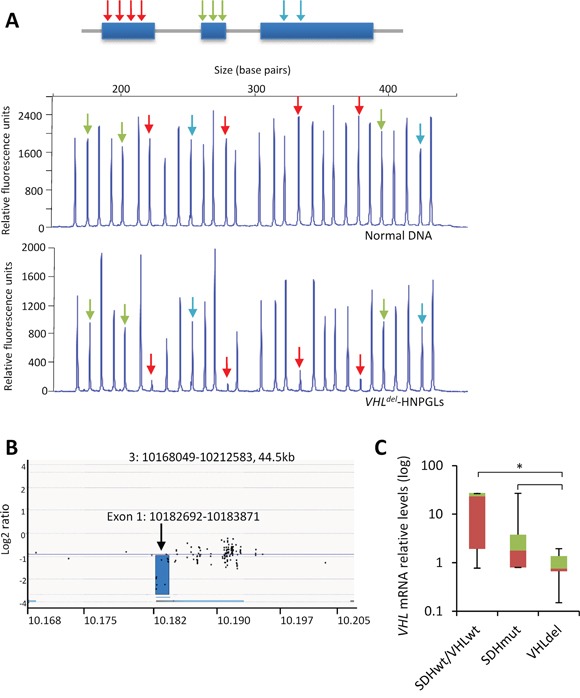
VHL exon 1 deletion in PGLs **A**. Upper panel, schematic representation of the structure of *VHL*. Arrows indicate exon location of the probe oligonucleotides included in the MLPA test. Middle and lower panels, capillary gel electrophoresis images depicting the MLPA assay for *VHL* in a normal control DNA and a tumor DNA. Red arrows indicate deleted exons. **B**. Array CGH screening of genomic DNA in a PGL sample with *VHL* exon 1 deletion as detected by MLPA assay. Profile is displayed as normalized log2 signal intensity ratios of each spot on the array to the genomic position at chromosome 3. **C**. RT-qPCR was performed for *VHL* in the indicated tumor types. Values are expressed as mean ratios ± SD. * *p* < 0.05 paired Student's *t* test.

*VHL* DNA copy number was analyzed by MLPA assay. We identified 6 samples exhibiting decreased copy number of exon 1 of *VHL* (hereafter *VHL^del^*-PGLs) (Table 1, Figure 6A) in tumor genomic DNA. This was significantly observed in PGLs lacking *SDHx* mutations (*SDHx^wt^*) (6/27, ~22%) but not in *SDHx* mutant (*SDHx^m^*)-PGLs (0/18) (*P*=0.02) and was not found in peripheral blood lymphocytes from any of these patients. These genetic defects were confirmed by an optimized quantitative PCR method in all tumors (data not shown), and by CGH microarray analysis in one tumor (Figure 6B). We next measured transcriptional levels of the *VHL* gene by RT-qPCR (n=36) to compare tumor samples with and without *VHL^del^*. As shown in Figure 6C, low *VHL* transcript levels were encountered in *VHL^del^*-PGLs as compared with PGLs lacking *VHL* or *SDHx* gene alterations (*SDH^wt^/VHL^wt^*) or tumors with *SDHx* mutations. Thus, 9 out of the 32 (28%) non-*SDHx* mutant PGLs included in this study carry *VHL* gene alterations (3 with *VHL* mutations -hereafter *VHL^m^*- and 6 with *VHL^del^*). This places this tumor suppressor among the most frequently altered genes described thus far in parasympathetic PGLs. Table 1 shows the association between the clinicopathological characteristics of the patients and the presence of *VHL^del^*. No association was found with age, location, functionality, multiplicity, or malignancy of tumors.

**Table 1:** Clinicopathological features of patients and association with VHL^del^

	n	del *VHL*	wtVHL*	*P*
**Age at diagnosis**				
≤40 years	23	3	20	0.697
>40 years	31	3	28	
UN^§^	3	0	3	
**Location**				
jugulotimpanic	28	4	24	0.693
vagal	8	0	8	
carotid body	19	2	17	
abdominal	1	0	1	
**Functional**				
no	53	4	49	0.472
yes	4	0	4	
**Single/Multiple**				
single	41	6	35	0.478
multiple	16	0	16	
**Benign/malignant**				
benign	55	5	50	0.067
malignant	2	1	1	
**SDHx mutation**^#^				
none	32	6	26	0.155
SDHB	8	0	8	
SDHD	16	0	16	
SDHC	1	0	1	

* wild type *VHL*;

§ unknown;

# germline mutations

**Figure 7:**
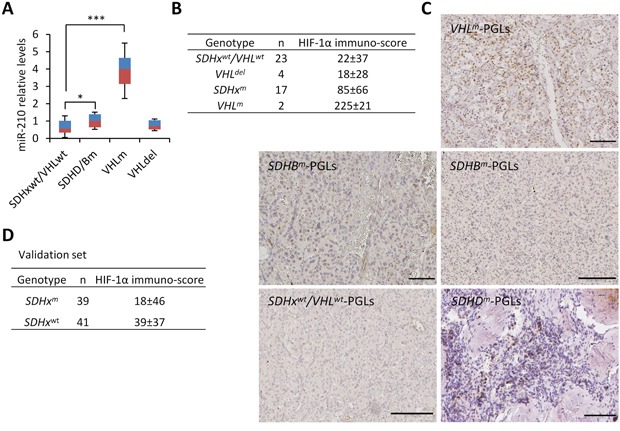
HIF-1α/miR-210 expression in SDHx- and VHL-mutant PGLs **A**. RT-qPCR was performed for miR-210 in the indicated tumor types. Values are expressed as mean ratios ± SD * *p* < 0.05, *** *p* < 0.001, paired Student's *t* test. **B and D**. Immuno-scores of HIF-1α in the tumor samples from patients with the indicated genotypes. **C**. Representative images of HIF-1α immunostainings in PGL samples from patients with *VHL^m^*, *SDHB^m^* or *SDHD^m^* or from a patient lacking *SDH* and *VHL* mutations (*SDH^wt^*/*VHL^wt^*).

We next analyzed the relative HIF-1α and miR-210 levels in tumors with specific genetic backgrounds: *SDHx^wt^/VHL^wt^*, *VHL^m^*, *VHL^del^*, and *SDHx^m^ (SDHB or SDHD)^m^*. miR-210 quantifications revealed that, as previously described, *VHL^m^*-PGLs expressed higher levels of miR-210 than *SDHx^m^-* or *SDHx^wt^/VHL^wt^*-PGLs (Figure 7A). However, *VHL^del^*-PGLs expressed low levels of miR-210 which were similar to those detected in *SDHx^wt^/VHL^wt^*-PGLs. In addition, *SDHx^m^*-PGLs expressed slightly higher, statistically significant, levels of miR-210 compared to *SDHx^wt^/VHL^wt^*-PGLs (p=0.045).

Then, we analyzed HIF-1α protein expression in PGL tumor tissues (n=46) by immunohistochemistry. Given that many of the samples showed non-homogeneous distribution of positive nuclei and distinct intensity of immunostaining, both, the percentage of positively stained cells and the relative intensity of immunostaining, were considered for HIF-1α quantification. Some of the tumors had been chemoembolized prior to surgery, but we did not find correlation between the percentage of positively immunostained cells and presurgical chemoembolization thus ruling out a “iatrogenic” effect of embolization not related to tumor biology. Overall, HIF-1α immuno-scores ranged from 0 to 240 with a mean value of 52 and median value of 17. Comparison of the immuno-scores in tumors revealed a gradient in the levels of HIF-1α protein spanning from very low (*SDHx^wt^/VHL^wt^*- and *VHL^del^*-PGLs) to low (*SDHx^m^*-PGLs) and high (*VHL^m^*-PGLs) expression levels (Figure 7B). The difference of immunostaining in *VHL^m^*-PGLs and *SDHx^m^*-PGLs lies in that the first has a high percentage of positive nuclei homogeneously distributed in the whole tumor section whereas the last takes on a patched appearance apparently not related to the distance to blood vessels (see representative immunohistochemical images in Figure 7C). In line with the miR-210 expression data, differences in HIF-1α immunostainings were statistically significant between *VHL^del^*-PGLs and *VHL^m^-* PGLs (p<0.0001) or *SDHx*-PGLs (p=0.03), but not between *VHL^del^*-PGLs and *SDHx^wt^/VHL^wt^*-PGLs. Differences were also statistically significant between *SDHx^m^*-PGLs and *SDHx^wt^*/*VHL^wt^*- (p=0.044).

Next, HIF-1α protein expression and *SDHx* genetic analysis was extended to a total of 112 parasympathetic PGLs (41 *SDHx^wt^/VHL^wt^*-PGLs and 39 *SDHx^m^*-PGLs all of them lacking *VHL* gene mutations) for validation. In addition to genetic analysis, the presence of *SDHx* mutations was analyzed by SDHB immunohistochemistry in 87 of the tumors given that negative immunostaining has been identified as a biomarker of *SDHx* mutations [22]. Indeed, our data showed that SDHB immunohistochemical negativity was significantly associated with the presence of mutations in any of the *SDHx* genes (p<0.0001). Comparison of the HIF-1α protein levels in tumors harboring or lacking *SDHx* mutations confirmed previously reported data revealing that *SDHx*-PGLs have a significantly higher percentage of HIF-1α positive cells than tumors lacking *SDHx* mutations (Figure 7D, p=0.008). No differences in HIF-1α protein levels were found, however, between *SDHD*- and *SDHB*-mutated PGLs/PCCs (p=0.999). Importantly, none of the *SDHx*-mutated tumors showed the very high percentage of positive nuclei that had been observed in *VHL*-PGL.

## DISCUSSION

In this report we have used cellular- and knockout murine-models and neuroendocrine PGLs to determine whether the HIF-1α and VHL proteins, implicated in the miR-210-mediated hypoxia/pseudohypoxia response *in vitro*, and the mitochondrial SDH complex, involved in HIF-1α stabilization, are essential for miR-210 expression *in vivo*. We demonstrated that ablation of *VHL* or *HIF1A* genes in neural crest-derived cells, from which the paraganglia originates, increases the basal levels of miR-210 (*VHL* knockout) or blunt the inducing effect of hypoxia (*HIF1A* knockout) on miR-210 indicating that these proteins are essential for the *in vivo* expression of miR-210 under hypoxic/pseudohypoxic conditions in paraganglia. By contrast, the mitochondrial SDH complex activity, related with tumorigenesis and supposedly involved in HIF-1α regulation [5], does not appear to have a direct effect on HIF-1α-dependent miR-210 over-expression but it favors it via a mechanism which has not been clearly defined as yet. Importantly, the data obtained in the murine/cellular models were corroborated by the use of human tumor materials containing known alterations of the *VHL* and *SDHx* genes. PGLs were chosen as tumor model because of their associations with mutations in those genes. The genotype-phenotype association studies revealed that the HIF-1α/miR-210 pathway is strongly activated in *VHL^m^*-PGLs, weakly activated in *SDHx^m^*-PGLs, and not activated in *VHL^del^*- and *SDHx^wt^*/*VHL^wt^*-PGLs. A functional link between defects of the *VHL* gene and activation of the miR-210 signaling pathway has also been demonstrated in other tumors such as PCCs, ccRCC and ovarian cancer [11, 23, 24]. However, the reported connections between HIF-1α and SDH in human tumors are more scarce and controversial.

The relative contribution of HIF-2α isoform in the development of PGL and miR-210 expression has not been explored in this report. As previously reported, nuclear HIF-2α protein expression is not detected in PGLs, irrespective of whether they carry *SDHx* or *VHL* mutations [11]. Therefore, although several lines of evidence suggest a predominant role for HIF-1α in miR-210 expression [11], the relative contribution of HIF-2α isoform in PGL development remains to be fully determined.

### Role of SDH on HIF-1α/miR-210 expression

Several studies have shown that the HIF-1α gene expression pathway is activated in *SDHx^m^*-tumors whereas others did not find such an association [11, 14, 16, 25]. Only a few of those studies have focused on correlation analysis between miR-210 expression and *SDHx*-mutations showing that overexpression of miR-210 was associated with *SDHx* mutations of PCCs, PGLs, and gastrointestinal stromal tumours [26, 27]. In the present report, we addressed the functional role of the SDH mitochondrial complex II components on the hypoxic/pseudohypoxic miR-210 up-regulation. By using RNA interference as well as conditional knock-out of *SDHD* via the Cre-Lox system, we demonstrate that absence of *SDHD* does not induce HIF-1α dependent miR-210 up-regulation. Similar data were obtained in cells with siRNA-induced *SDHB* deficiency. We also show that this effect was mimicked by TTFA, a SDH inhibitor that blocks the mitochondrial electron flow by binding to the ubiquinone docking sites at the SDH complex [28]. Conversely, PGL-derived cells carrying *SDHC* or *SDHB* mutations do show mild activation of HIF-1α and miR-210. These data agree with those observed in PGLs, i.e. HIF-1α/miR-210 levels were found higher in *SDHx^m^*-PGLs as compared to *SDHx^wt^*-PGLs although lower than those detected in *VHL^m^*-PGLs. Given that the data obtained in murine and cellular models support that SDH loss of function do not directly activate the HIF-1α pathway, that HIF-1α nuclear accumulation is only observed in about 38% of tumor cells in *SDHx^m^*-PGLs, and that these tumors show mild miR-210 over-expression, we postulate that HIF-1α up-regulation in these tumors is a late event that it is favored by *SDHx* mutations but is triggered by still undefined biochemical or genetic event/s. Mitochondrial complex II activity catalyzes the oxidation of the metabolite succinate, which is involved in HIF-1α hydroxylation, to fumarate. The electrons generated in this reaction are then channeled, in a not completely understood pathway, to ubiquinone, which is reduced to ubiquinol by the succinate:ubiquinone oxidoreductase activity of the complex. Electrons provided by succinate oxidation may leak out from complex II and lead to the formation of sub-lethal levels of ROS. Although existing literature includes many contradictory findings, mitochondrial production of ROS is involved in regulating hypoxia-induced HIF-1α protein stabilization [29, 30]. Thus, other possibility is that *SDHx* mutations favor an intracellular microenvironment rich in succinate and/or ROS that only when they reach a threshold level may trigger HIF-1α protein accumulation. This provides an explanation for the published contradictory data regarding the link of SDH dysfunction and HIF-1α. Further biochemical studies will be required to unravel the specific mechanisms involved in regulation of the HIF-1α/miR-210 axis by pathogenic mutations on *SDHx* genes.

### Role of VHL on HIF-1α/miR-210 expression

Another major finding of this study is the identification of recurrent deletions of exon 1 of the *VHL* gene (19% of tumors) in PGLs developed in patients lacking germline *SDHx* mutations. Previous studies revealed that losses of *VHL* are rare in PGLs [31–33] but not in renal cell cancer or *VHL*-related PCCs. Thus, this is the first description of somatic *VHL* gene exon deletions in parasympathetic PGLs. Nevertheless, the phenotypic consequences of *VHL* gene alterations seem to be heterogeneous depending on the type of defect. As stated above, we definitively demonstrated that PGLs carrying *VHL*^m^ are grossly defective with respect to HIF regulation and induce a strong activation of miR-210. However, we also discovered that partial loss of *VHL* in tumors is not accompanied by HIF-1α activation or miR-210 over-expression. In accord with these findings, miR-210 over-expression was not detected in the adrenal medulla from heterozygous *VHL* +/- mice. Absence of correlations between phenotypes induced by loss of gene expression versus gene mutation has been demonstrated in zebrafish, mouse and *Arabidopsis* [34–37]. In this last setting, Rossi et al, provided an explanation for those observations [38]. They found that deleterious mutations, but not gene knockdown, trigger compensatory responses that preclude observation of phenotypes similar to those induced by gene knockdown. Thus, we cannot rule out the possibility that loss of *VHL*, in contrast to *VHL* mutations, activates compensatory networks to cushion the deficit. It is also possible that *VHL* genetic defects may represent “passenger” events not relevant to carcinogenesis. Alternatively, *VHL* partial deficiency may activate HIF-independent pathways which may be relevant to its tumour suppressor function [39, 40]. Ultimately, it can also be speculated that *VHL*-reduced expression versus *VHL*-gene mutations may elicit different effects on VHL stability and/or protein-protein interactions and lead to a distinct non canonical HIF-1α-related biochemical outputs [41]. Prior to this study, microarray gene expression studies had been performed in PGLs including a few of the *VHL^del^*-PGLs identified in this study [11]. Re-analysis of the microarray data has revealed a constrained list of HIF-1α-related deregulations in *VHL^del^*-PGLs which was limited only to genes involved in angiogenesis and extracellular matrix remodeling, molecular processes heavily related to cellular proliferation and differentiation. These data suggest that the HIF-1α protein levels reached under these circumstances, if any, are not sufficient for induction of miR-210 expression but they are possibly enough to induce other HIF-1α-related genes. If confirmed with broader studies, the observed genotype-phenotype associations would argue in favor of *VHL* as a driver gene in sporadic PGLs. Further genetic and cell-based assays are required to fully understand the functional significance of the observed genotype-phenotype associations.

We also provide experimental evidences supporting that a mutant VHL protein, F76del, acts as a dominant-negative inhibitor of the wild-type VHL by inducing the pro-tumorigenic HIF/miR-210 pathway even in the presence of wild type VHL. This study was of interest because many *VHL* mutations involved in human cancer, especially those found in PGLs, are not always accompanied by loss of the wild type allele. Thus, the question remained whether mutant VHLs are also defective for HIF-1α regulation and able to up-regulate miR-210 in the presence of wild type VHL. We selected the F76del mutant VHL to address this issue because this mutation has been associated with distinct types of cancer such as renal cell carcinomas, PCCs and PGLs [42]. Previous association studies had shown that mutations of *VHL* in tumors correlate with miR-210 over-expression [11, 23, 26, 27]. In addition, a disturbance of the coupling between HIF-1α and mutants VHL, including F76del, has been experimentally confirmed by other authors using a series of *in vitro* assays [43]. However, most of those studies were performed in *VHL*-deficient cells in which the *VHL* gene expression was restored by transfection of the mutated *VHL* gene [44]. Thus, other than association studies, direct evidences for a role of F76del on miR-210 expression was lacking and it was not known whether loss of the wild type allele was required for the activation of the pro-tumorigenic HIF pathway by the mutant VHL.

In summary, the analyses of different pseudo-hypoxic biomarkers in different settings are fully consistent and strongly suggest that the HIF-1α-related expression profile may be finely adjusted by SDH and VHL. *VHL* mutations are associated with a strong HIF-1α-dependent miR-210 over-expression and accumulation of HIF-1α in most tumor cells whereas *SDHx* mutations are associated with mild increase of miR-210 and the presence of a heterogeneous, HIF-1α-positive and HIF-1α-negative, tumor cell population. Thus, accumulation of HIF-1α protein seems to be an early event in the tumorigenic pathway led by *VHL* mutations but a deferred episode in tumors harboring *SDHx* mutations. The question remains as to whether the distinct pseudo-hypoxic landscapes identified in PGLs translate into different clinical phenotypes as, for example, the multiplicity of tumors, the risk of developing PGLs of sympathetic or parasympathetic origin, or the different tendency to develop metastasis [45]. Certainly, the data obtained in the present study will acquire a more general clinical relevance in cancer pathogenesis and will pave the way for the understanding of the effect of genetic events on pseudo-hypoxic signaling and its role in the pathogenesis of tumors, a knowledge that will allow the use of optimal and individualized therapeutic targeting of tumors harboring these genetic changes.

## MATERIALS AND METHODS

### Tumor specimens

Tumor samples were obtained from 57 patients with parasympathetic PGLs, diagnosed and treated between 1996 and 2012 in the Hospital Universitario Central de Asturias and Hospital Gregorio Marañón (Table 1). For validations, independent set of tumors from 119 patients was used. For RNA/DNA-based studies, fragments containing more than 70% tumor cells were obtained from the core of the tumor. Tumor specimens were snap-frozen at time of surgical resection and stored at −80 °C in RNAlater (Ambion) until processed. All but two tumors had benign clinical behavior. One of the patients with malignant PGL developed regional lymph node metastasis and died six years after diagnosis. Informed consent was obtained from all patients. The study was approved by the Ethical Committee of the Hospital Central of Asturias and the methods were carried out in accordance with the approved guidelines.

### Cell culture

SCC40 cell line, derived from larynx squamous cell carcinoma, was kindly provided by Dr. R. Grenman (Department of Otolaryngology, University Central Hospital, Turkey, Finland). SCC40, 786-O and RCC4 cells were grown in DMEM supplemented with 10% fetal bovine serum, 100 units/mL penicillin, 200 μg/mL streptomycin, 2mmol/L L-glutamine, 20 mmol/L HEPES (pH 7.3), and 100 μmol/L nonessential amino acids. Cell line authentication was performed by array CGH [19]. Growth curve analysis was performed regularly, and no phenotype changes were observed through the duration of this study. To avoid cross-contamination and phenotype changes, these cell lines have not been maintained in long-term cultures. Mouse embryonic fibroblasts (MEFs) and baby mouse kidney (BMK) cells were obtained from *SDHD*-ESR mice (*SdhD*−/−) and their homozygous *SdhD*^*flox/*+^ (*SdhD*+/+) and heterozygous *SdhD*^*flox*/−^ (*SdhD*+/−) littermates and cultured in medium supplemented with 4-hydroxy-tamoxifen for 4 or 24 hours as previously described [20].

### Genetic testing

Genomic DNA was isolated using the QIAmp DNA Mini kit (Qiagen, Inc., Chatsworth, CA) and subsequently treated with RNase A (1unit/mL) at 37°C for 5 minutes. Mutation analysis was performed by direct sequencing as previously described [16]. *VHL* gene was analyzed for the presence of large deletions in tumor DNA using the Multiplex Ligation-dependent Probe Amplification (MLPA) (SALSA MLPA P016, MRC Holland, The Netherlands) method as recommended by the manufacturer. Analysis of the amounts of the MLPA PCR products per gene was done on an ABI PRISM 3100 Genetic Analyzer platform (Applied Biosystems) according to the manufacturer's instructions. Each gene peak area was divided by the sum of the control peak areas for that sample. These relative areas were then compared with the corresponding average relative area obtained from three normal mucosa samples, thus creating a ratio (case/normal). The mean ratio obtained from at least two independent MLPA reactions for each sample was considered the final result. For a normal mucosa, this ratio was expected to be 1. DNA copy numbers for *VHL* gene were confirmed by real time PCR and Array Comparative Genomic Hybridization (array CGH).

Real time PCR was performed following as previously described [46]. Relative DNA quantity was determined using the delta-C_t_ formula. For normalization of the relative quantities, the *VHL* copy numbers were divided by the geometric mean of the reference *GPR15* copy number.

Screening of genome-wide copy number variants was carried out by array-based CGH using the OncoNIM® Familial Cancer platform, a 60 k Agilent based custom array-CGH (Nimgenetics; Madrid, Spain). This custom array covers the whole genome with a median spatial resolution of 1 probe per 150 kb, with high density coverage in 20 genes related to familial cancer (100 bp median spatial resolutions for these genes). Hybridizations were performed according to the manufacturer's protocols. A commercially available male DNA sample (Promega, Madison, WI, USA) was used as reference DNA. Microarray data were extracted and visualized using the Feature Extraction Software v10.7 and Agilent Genomic Workbench v.5.0 (Agilent Technologies, Santa Clara, CA) using ADAM-2 (windown 0.5Mb, A=10) as aberration detection statistic. Genomic build NCBI37 (Hg19) was used for delineating the genomic coordinates of the detected CNVs.

### RT-qPCR

Total RNA was isolated with the *mir*Vana™ miRNA Isolation Kit (Ambion) according to the manufacturer's instructions. cDNA synthesis and real-time PCR were performed as previously described [18]. Briefly, real-time PCR reactions were performed by using SYBR Green PCR Master mix (Applied Biosystems) and the thermocycler conditions recommended by the manufacturer. Each sample was analyzed for cyclophilin A mRNA to normalize for RNA input amounts and to perform relative quantification. Primers were designed using the computer program Primer Express (Applied Biosystems). Primers were as follows: *VHL*, forward 5’-CCCAGGTCATCTTCTGCAAT-3’- and reverse, 5’-ACATTTGGGTGGTCTTCCAG-3’; cyclophilin A, forward, 5’-CATCTGCACTGCCAAGACTGA-3’ and reverse, 5’-TTGCCAAACACCACATGCTT-3’; *CA9*, forward, 5’- CGCTGAGGAAGGCTCAGAGA-3’ and reverse 5’- GGCAGGAGTGCAGATATGTCC-3’; *EGLN3* 5’-CCGGCTGGGCAAATACTATG-3’ and reverse 5’- GATAGCAAGCCACCATTGCC-3’. Melting curve analysis showed a single sharp peak with the expected Tm for all samples. mRNA relative quantities were obtained using the 2ΔΔCt method. Individual TaqMan assay (Applied Biosystems) was used to analyze the expression of the mature human miR-210. 10 ng of total RNA was used in the RT reaction and the transcribed cDNA was then used for subsequent PCR amplification using TaqMan 2X Universal PCR Master Mix, No AmpErase UNG (Applied Biosystems) as described by the manufacturer. RNU44 expression was assayed for normalization. All reactions were performed in triplicate, and relative miRNA expression was normalized against endogenous controls using the comparative delta-delta CT method.

### *VHL* and *HIF-1α* knockout mice

In order to delete *HIF1A* or *VHL* production specifically in neural crest derived cells, we used conditional knockout mice carrying floxed alelles of *HIF1A* [47] or *VHL* [48] genes crossed with transgenic mice carrying a tamoxifen-inducible CRE recombinase whose expression is driven by the promoter of the intermediate neurofilament Nestin (Nes-CreERT2 mice) [49]. The resulting strains undergo deletion of *HIF1A* or *VHL* specifically in neural crest-derived tissues expressing nestin protein and only after tamoxifen treatment. We injected 14 mg/kg of tamoxifen to the 8-weeks old mice in 3 doses in a week. *HIF1A* −/− or control mice *HIF1A* +/+ were put in a hypoxic chamber at 10% O_2_ for 1 month. Animals were sacrificed and adrenal medulla was dissected. All experiments were performed in accordance with institutional guidelines and were approved by the animal ethics committee of the University of Oviedo.

### Site-direct mutagenesis

Site-direct mutagenesis was performed in pcDNA3.1 HA-*VHL* construct, kindly provided by Holger Moch (University Hospital Zurich, Institute of Surgical Pathology) using the GeneArt Site-Directed Mutagenesis System (Life Technologies) in order to introduce F76del mutation in the *VHL* cDNA. Gene amplification and bacterial transformation followed the protocol described by the manufacturer and the mutated DNA sequences were screened by nucleotide sequencing. Primers used to generate the mutations were: 5’-CCTCCCAGGTCAGCAATCGCAGTCCGC-3’ and 5’-GCGGACTGCGATTGCATATGACCTGGGAGG-3’.

### siRNA treatment

siRNA duplex oligonucleotides were purchased from Dharmacon Research (Lafayette, CO, USA). siCONTROL Nontargeting pool (Dharmacon) were used as control siRNA. SCC40 cells were transfected with 35 pmol/ml siRNAs using Lipofectamine 2000. mRNA analysis analyses revealed a substantial inhibition of SDHB or SDHD expression 48–72 h after transfection. The transfected cells were used for subsequent experiments within that interval of time.

### Western blot analyses

Protein extracts were obtained from SCC40 cells at 80–90% confluence as previously described [18]. Equal amounts of proteins were fractionated on SDS–PAGE and transferred to PVDF membranes. Membranes were probed with anti-HIF-1α, (Becton Dickinson Transduction Laboratories, Erembodegem, Belgium), or anti-β-actin (Sigma-Aldrich, St Louis, MO, USA) antibodies at 1:1000 dilutions. Bound antibodies were detected using enhanced chemiluminescence reagent (Life Technologies) according to the protocol of the manufacturer. Densitometry of Western blot analyses was conducted with ImageJ analysis software.

### Immunostainings

HIF-1α expression was evaluated by immunohistochemistry in tissue sections from a retrospective cohort of 46 surgically resected PGLs as previously described [16]. Following published recommendations [50], both, the percentage of immunostained cells and the intensity of staining were used to quantify HIF-1α protein expression. Staining intensity was scored from 0 to 3+. Data were reviewed independently by 3 investigators. For immunofluorescence analysis, Alexa555-conjugated anti-mouse IgG was used as secondary antibody and cells were pictured using Zeiss AxioObserver Z1 microscope with AxioCam MRM and ApoTome 2 (Carl Zeiss, Germany).
